# End to End Colonic Content Assessment: ColonMetry Application

**DOI:** 10.3390/diagnostics13050910

**Published:** 2023-02-28

**Authors:** Bernat Orellana, Eva Monclús, Isabel Navazo, Álvaro Bendezú, Carolina Malagelada, Fernando Azpiroz

**Affiliations:** 1 Visualization, Virtual Reality and Graphics Interaction Research Group, UPC-BarcelonaTech, 08034 Barcelona, Spain; 2 Digestive Department, University Hospital General de Catalunya, 08190 Barcelona, Spain; 3 Digestive System Research Unit, University Hospital Vall d’Hebron, 08035 Barcelona, Spain; 4 Departament de Medicina, Universitat Autònoma de Barcelona, 08193 Bellaterra, Spain; 5 Centro de Investigación Biomédica en Red de Enfermedades Hepáticas y Digestivas, Ciberehd, 28029 Madrid, Spain

**Keywords:** magnetic resonance imaging, colonic content, colon segmentation, intestinal biomass, intestinal gas, functional digestive disorders

## Abstract

The analysis of colonic contents is a valuable tool for the gastroenterologist and has multiple applications in clinical routine. When considering magnetic resonance imaging (MRI) modalities, T2 weighted images are capable of segmenting the colonic lumen, whereas fecal and gas contents can only be distinguished in T1 weighted images. In this paper, we present an end-to-end quasi-automatic framework that comprises all the steps needed to accurately segment the colon in T2 and T1 images and to extract colonic content and morphology data to provide the quantification of colonic content and morphology data. As a consequence, physicians have gained new insights into the effects of diets and the mechanisms of abdominal distension.

## 1. Introduction

**Figure 1:**
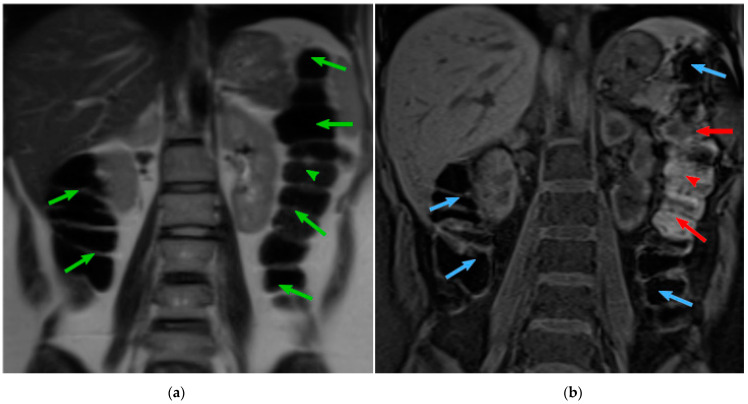
(**a**) MRI T2 coronal slice showing ascending colon and descending colon (green arrows). (**b**) The same area in the T1-FS modality. Arrows are colorized depending on the contents: blue arrows point to gas and red to feces. Courtesy of [7].

About half of the patients in gastroenterological practice complain of digestive symptoms without detectable abnormalities by conventional diagnostic tests. In a large proportion of these patients, specifically those with intestinal functional disorders, their symptoms seem related to colonic dysfunction and abnormal intraluminal contents; however, the evaluation of the volume and distribution of solid and gas content in the colon is fraught with methodological constrains. Abdominal imaging by means of computer tomography (CT) has shed some light into the physiology of colonic handling of contents and disease [1,2,3,4,5]. Magnetic resonance imaging (MRI) has recently become an alternative technique to CT, since it provides an appropriate visualization of soft tissues while avoiding the exposure to ionizing radiation used in CT [6]. The MRI acquisition technique allows the modification of the signal emitted to acquire the different tissues presented in the body. The MRI modalities commonly used for the analysis of colonic content are T2-weighted (T2) and T1-weighted fat-SAT (T1-FS). However, those techniques, by themselves, do not allow the measurement of the colonic content. T2 sequence offers an optimal visualization of the colon boundaries, contrasting the colon lumen (dark voxels) against the fat around the colon (bright voxels) (Figure 1a). T1-FS sequence (Figure 1b) provides visual discrimination of the colonic content: feces are bright regions, while colonic gas is dark. Therefore, the information found in T1-FS and T2 images is complementary and both modalities are required to obtain a quantitative assessment of colonic morphology and content. To study the gut in normal conditions, it is necessary to acquire the abdominal images without preparation, e.g., administration of drugs or contrast.

In general, MRI scans are harder to segment than CT scan images due to the lack of fixed correspondence between tissues and intensities and the incidence of noise and artifacts on the images [8]. T2 modality displays the colon as a dark region contrasted with bright surrounding fat, but its appearance may suffer large intensity and texture variations along its tract, mainly caused by the presence of colonic content. Regarding T1-FS, this modality allows for the proper discrimination of fecal content: feces are bright regions while colonic gas is dark. However, fat tissues are also dark, almost indistinguishable from the adjacent colon gas. In consequence, colonic boundaries in T1-FS are not visible in regions with little presence of faces. Since clinical research involves numerous studies to be analyzed, and current colon segmentation algorithms are time-consuming for the specialists, the clinical practice would benefit from a higher degree of automation of colon segmentation algorithms.

Previous studies evidenced the practical limitations of manual segmentation of intestinal imaging obtained by different MRI modalities [9,10,11]. In this regard, by a close interaction between physicians and computer scientists, our group developed original tools devoted to colon segmentation in T2-weighted half-Fourier acquisition single-shot turbo spin echo (HASTE) (T2) and T1-weighted VIBE Fat-Sat (T1-FS) sequences. Ceballos et al. [12] developed an end-to-end application for colonic content assessment from T2 and T1-FS sequences, allowing the quantification of the colon volume from T2 images and the fecal volume content from T1-FS images. Moreover, some shape properties of the colon were also computed (perimeter and length). The application works on a 3D volume model built from the MRI images. Our application provided a seed region growing mechanism combined with the ability to add stop markers to prevent leaking. The colon segmentation obtained in T2 images was transferred to T1-FS images using a non-rigid semi-automatic registration method; however, a manual specialist validation evidenced that only the fecal content was properly identified. Once both modalities have been segmented, an automatic classification method determined the amount of fecal content inside the colon. This pipeline has been used in different clinical experiments [5,13], but it requires a relatively long time that the specialist had to dedicate (40 min by scans (T2 and T1-FS)), so there was space to improve basically in the two segmentation processes.

Following this line, we developed a quasi-automatic segmentation algorithm for the colon on T2 images based on an unsupervised clustering algorithm guided by a probabilistic measure extracted from a training set [7,14]. The process drastically reduced the specialist interaction effort compared to other state-of the-art solutions (5 min by scan). Focusing on the automation of colon segmentation in T1-FS, we subsequently developed a specialized registration algorithm that provides an accurate and fast colon segmentation in T1-FS using as an input the colon segmentation from T2 images ([15]: under review process). From the first pipeline, many improvements have been developed to facilitate obtaining adequate colonic segmentation. However, it is important to note that the specialist always needs to supervise and validate the results after T2 and T1-FS colon segmentations have been computed, and this validation is not straightforward. For some areas, the analysis of a single modality may be confusing, and the validation may require the simultaneous visualization of both modalities. For instance, the cecum and sigma segments are very difficult to distinguish from the small bowel in T2 modality, but the detection (and validation process) is substantially simplified by simultaneous inspection of T1-FS and T2 images. To address this medical problem, the objective of the present study was to develop a complete end-to-end quasi-automatic framework to segment the colon in T2 and T1-FS MRI sequences for quantitative evaluation of colonic morphology and the discrimination between the gas and non-gaseous (solid) colonic content. In this paper, we present a novel interactive module to visualize both segmentations synchronously, to facilitate the validation of the inter-modality coherence between both segmentations, and its evaluation by domain experts. Finally, although some modules have been previously validated, here we present a complete medical validation and discussion about the integrated segmentation and interaction techniques for all the components of the pipeline.

## 2. Materials and Methods

**Figure 2:**
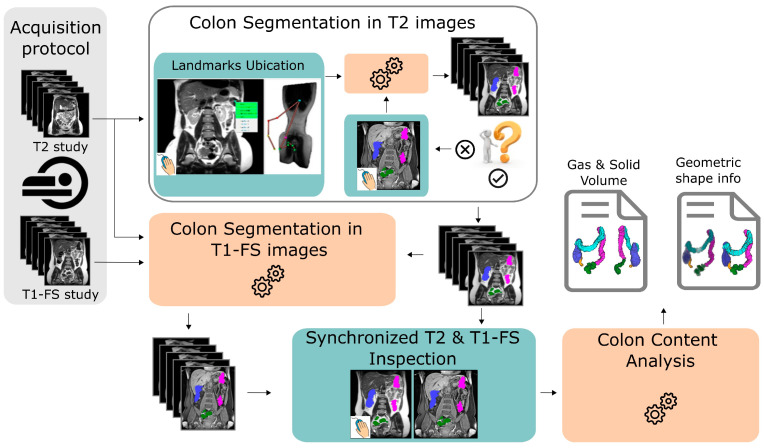
Overview of the system: the left column shows the algorithm’s inputs: T1-FS images and T2 images. First, the specialist provides a minimal set of 5 anatomical reference points along the colon path. From this information, the system automatically computes the colon segmentation in T2 images. If the obtained segmentation is not accurate enough, the specialist can add refinement markers with the objective to automatically correct misclassified regions. Once the colon segmentation has been validated by the medical expert, the system automatically computes the colon segmentation in T1-FS. Then, the new module allows visualizing both segmentations simultaneously and synchronously, to validate the inter-modality coherence between them. Finally, the system automatically computes different properties of the colon shape and colonic content information.

The application’s framework is shown in Figure 2. The patient is scanned twice to obtain T1-FS and T2-weighted images. Once the images are captured, the specialist provides a minimal set of 5 anatomical reference points along the colon in T2 images. From this information, the system automatically computes the colon segmentation in T2 images. If the obtained segmentation is not accurate enough, the specialist can add refinement markers with the objective to automatically correct misclassified regions (see Section 2.1). Next, the colon segmentation in T1-FS images is carried out using an automatic approach that uses the colon segmentation previously calculated in T2 images (see Section 2.2). Then, the medical expert can interactively visualize both segmentations to validate them (see Section 2.3). Finally, the non-gaseous and the gaseous colonic content and different properties of the colonic morphology are quantified using both segmentations (see Section 2.4). Each module of the system is able to provide a 3D visualization of the colon, which helps to understand the colon anatomy.

### 2.1. Colon Segmentation in T2 Images

In Ref. [7] we presented a novel accurate colon segmentation algorithm from unprepared MRI in T2 images with a low user effort. The process only required the specialist to provide a minimal set of five anatomical reference points (*marker points*) along the colonic trajectory to guide the segmentation algorithm. These points determined the start and end of each of the colon segments: ascending, transverse, descending, and pelvic. Depending on the complexity of the colonic shape, more points may be required to ensure a correct segmentation result. From this information, the algorithm automatically computed the colonic segmentation. The algorithm is founded on the estimation of the colon medial path and the usage of a probabilistic model for tissue classification based on the MRI intensities and the colon medial path distance. A probability model guides a progressive segmentation process based on a multigrid architecture using a 3D graph-cuts algorithm. This multigrid architecture provides computational scalability to the solution.

### 2.2. Colon Segmentation in T1-FS Images

In Ref. [15] we presented an automatic colon segmentation algorithm for T1-FS images. The algorithm requires three inputs, the abdominal T2 and T1-FS images, and the colonic segmentation in T2 modality explained in Section 2.1. From these inputs, the algorithm computes a non-rigid transformation (*RegisterTrans f_T2_*_→*T1−FS*_) to obtain the spatial correspondence between T2 and T1-FS images. Next, an iterative registration process accurately adapts the initial registered colon to T1-FS images. This iterative registration phase is driven by a mesh deformation process based on a T1-FS content probabilistic model and mechanisms for preserving the T2 colon shape.

### 2.3. Synchronized T2 and T1-FS Inspection

As mentioned in Section 1, there may be confusing areas of the colon that require the simultaneous visualization of both modalities to understand the colonic anatomy. Therefore, once both segmentations have been computed, their simultaneous visualization and inspection allows the specialist to analyze, validate, and, if it is needed, to correct the results.

To achieve this, we used the non-rigid registration transformation computed in Section 2.2 to obtain the spatial correspondence between T2 and T1-FS images. Specifically, given a point P in a T2 image, we can compute its correspondence point in the T1-FS space as: *P_T_*_1*−FS*_ = *RegisterTrans f_T_*_2*→T*1*−FS*_(*P*). Similarly, from a point P in a T1-FS image, its correspondence point in the T2 space is: *P_T_*_2_ = *RegisterTrans f_T_*_1*→FS-T*2_(*P*).

**Figure 3:**
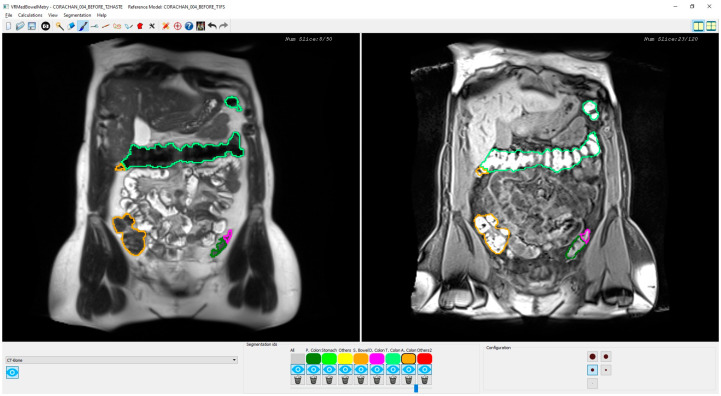
Snapshot of the synchronized T2 and T1-FS module. On the left side, an original T2 image is shown with the colon segmentation outlined using a different color for each segment. On the right side, the same visualization scheme is used with its corresponding original T1-FS image.

Figure 3 shows a snapshot of the application interface in which two T2 and T1-FS synchronized images of a segmented colon are visualized in two different windows. Every time the specialist clicks on a pixel of one image (T2 or T1-FS) the system automatically computes its corresponding pixel position in the other image (T1-FS or T2) by transforming the pixel position using the formula previously presented. Moreover, using the mouse wheel, the user can navigate forward and backward around the images in a synchronous fashion following the same scheme: when the specialist moves the mouse wheel on one window, both views are updated accordingly to display the same location. The window directly affected by the wheel is moved proportionally to the wheel displacement, whereas the movement of the other window has to be calculated using the transform previously presented.

The main novelty of this inspection functionality is that, to our knowledge, it is the first one able to allow synchronous navigation of both MRI sequences considering the deformation suffered in the acquisition process. Note that the original MRI images are visualized without altering them, but the interactive navigation allows computing the corresponding image using the non-rigid transformation matrix. Moreover, the synchronized inspection facilitates a 3D mental reconstruction of the colon in both MRI modalities, which is helpful to validate the segmentation result in both sequences, and the medical expert can modify the colon segmentation, if needed, either in T2 or T1-FS independently. The 3D simultaneous visualization of both segmentations is also possible. However, performing corrections in the 3D space is quite complex and medical specialists prefer the 2D synchronized visualization of T2 and T1-FS images since it provides detailed information about the anatomical structures around the colon and facilitates the validation of the boundaries in the automatically generated segmentations.

### 2.4. Colon Content Analysis

Once the segmentation of the colon has been obtained in both sequences and has been validated by the specialist, the computation of the colonic content can be performed. This process consists of the classification of pixel intensities according to the percentage of solid content they represent. The solid percentage computation is based on a k-means clustering approach (details in [12]). Apart from fecal and gas content, other parameters related to the shape of the colon (length, perimeter, and radius) could also be computed from the colonic segmentation in T2 images (details in [16]).

### 2.5. Acquisition Protocol

The proposed framework has been used in independent clinical research studies. In all the experiments, each time a subject was scanned, two coronal image series of the abdomen were obtained: a T2-weighted HASTE (T2) sequence during two apneas of approximately 20 s each and a T1-weighted VIBE Fat-Sat (T1-FS) sequence in one apnea of 12 s. The imaging examinations were performed using two different 1.5-T MR imaging systems (Aera; Siemens Healthcare, Erlangen, Germany and Ingenia; Philips Medical System, Andover, USA) with abdominal coils wrapped around the abdomen. The subjects were placed in prone position. The specific acquisition protocols for the different studies were also different for each MR system (see [4,5,13] for a detail description). Images were obtained without oral or intravenous contrast, colonic infusion of gas or liquids or administration of antiperistaltic drugs. All images were codified and analyzed blind concerning the source, acquisition, and any preceding intervention by two trained physicians in MRI images under the supervision of an expert radiologist.

## 3. Results

In this section, the experiments carried out to evaluate each module of the overall pipeline are presented. The experiments tested the accuracy obtained by the segmentation algorithms, the computational and the user interaction cost, and the usability of all the modules.

### 3.1. Validation of the Colon Segmentation from T2 and T1-FS Images

**Figure 4:**
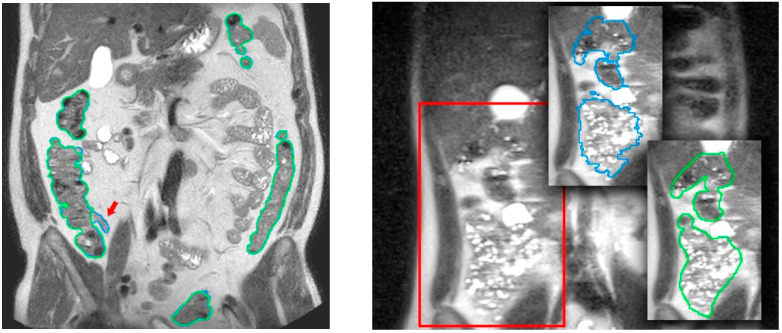
Comparison of segmentation results (in blue) with a ground truth manual colon segmentation (in green) in T2 images. In the left image, the red arrow marks a small ileum region wrongly assigned to colon by the automatic algorithm. In the right image, a highly challenging area even for the specialists (outlined in red) is shown. Courtesy of [7].

The validation of the colon segmentation from T2 images was presented in [7]. In total, 52 scans were used in the accuracy experiment: 30 scans from 15 healthy participants (8 women, 7 men) and 22 scans from 11 patients (8 women, 3 men) with irritable bowel syndrome (IBS); participants had a normal body mass index or were overweight (body mass index between 18.5 and 29.9 Kg/m^2^), i.e., no underweight or obese subjects were included. The assessment of the algorithm accuracy was performed by comparing the segmentation results to the ground truth (specialist manual segmentation) using the dice similarity coefficient (DSC). The achieved accuracy was (*µ_DSC_* = 0.85, *σ_DSC_* = 0.06), demonstrating its suitability for clinical usage. Figure 4 compares results of the colon segmentation computed by the algorithm with respect to a ground truth segmentation manually performed by the medical expert. In the left image, the medical expert did not realize that a small ileum region had been wrongly identified as colon by the segmentation algorithm. This misclassification was probably due to a similar pixel intensity in both the ileum and the adjacent colon, which made it very difficult to identify the colon in that area. As explained in Section 3.2, a simultaneous visualization of the T1-FS images could have helped him to fix this wrong identification. From the point of view of performance, the average execution times (in minutes) for all the volumes involved in the experiment were in the range 5.7 ± 1.16 min, with a maximum peak of 8.5 min, and the memory usage was below 2.5 GB. Focusing on the usability of the process, the experiments demonstrated that this approach could be considered as low demanding in terms of user time (on average 5′ of usage time). Compared to the initial approach presented in [12], usage times have been reduced by 80%.

Regarding the validation of the colon segmentation in T1-FS images, the algorithm accuracy was evaluated by comparing the automatic segmentation results to manual segmentations of the colonic fecal content (taken as ground truth) performed by the specialist (in [15] the validation experiments were presented). Note that the specialist only segmented the fecal content because the complete segmentation was too demanding and almost impossible in areas where no fecal content was presented (areas with gas).

**Figure 5:**
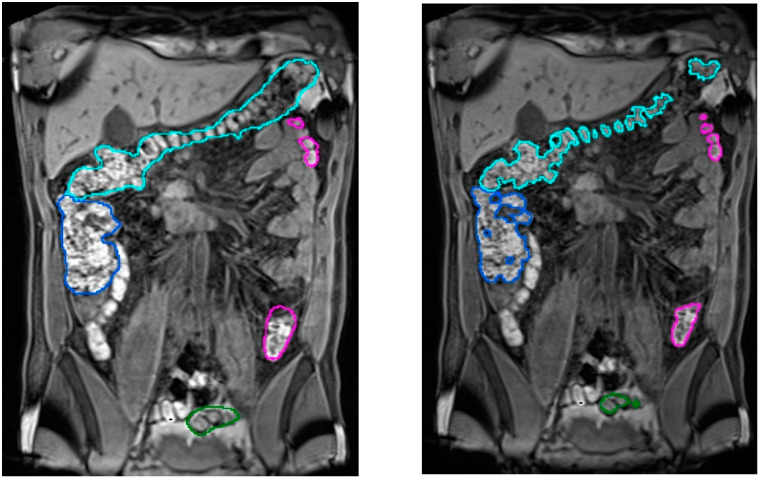
Comparison of segmentation results in a T1-FS coronal slice. In the **left** image, the original slice visualized with the corresponding automatic colon segmentation outlined using a different color for each of its segments. In the **right** image, the same coronal slice is visualized with the corresponding manual identification of the fecal content, used as a ground truth in the validation process.

In total, 90 scans obtained from 35 healthy volunteers (8 women, 27 men; 20–28 Kg/m^2^ body mass index range) were used. The quantitative validation metric, named R, was defined as the percentage of ground truth fecal content inside the colon segmentation result. The achieved accuracy was (*μ_R_* = 96.2%, *σ_R_* = 4.2), demonstrating its viability for clinical usage (see Figure 5). The mean execution time was 9 min without any human intervention.

### 3.2. Validation of the Synchronized Navigation in T2 and T1-FS Images

**Figure 6:**
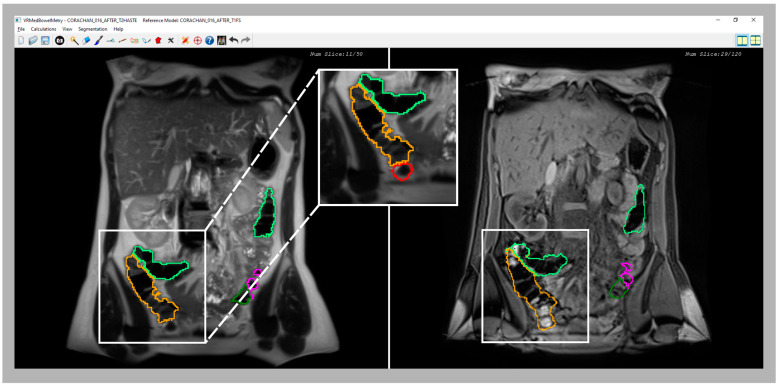
Comparative images of a confusing area of the cecum in T2 (**left** image) and T1-FS (**right** image) sequences. Note that in the T2 sequence, part of cecum was not included in the initial segmentation because it was confused with the small bowel; a zoom (**central** image) provides a better visualization of the non-included area (red line). However, in the synchronized visualization with the T1 sequence (**right** image), the feces facilitate the cecum demarcation. Therefore, using the new inspection module, the medical specialist should be able to correct and include the cecum in the final segmentation.

As shown in previous sections, a synchronized visualization of T1-FS and T2 sequences is required to validate the coherence between both colon segmentations and, also, to correct the results in some confusing zones (see an example in Figure 6).

The validation of this module consisted of the visual analysis of some colon segmentation results by medical experts. We specially chose those cases where the isolated validation of T2 and T1-FS segmentation showed some small differences.

In all the analyzed cases, when the specialist detected a complex area where the colon was difficult to identify using only one sequence (T2 or T1-FS), the analysis of the same area in the other sequence helped him to decide. In general, these confusing areas were located in the T2 images, especially where the small intestine was very close to the colon and their pixel intensities were similar (see Figure 6). The new module facilitates and gives security to the specialists at the time of determining the belonging of the area to the colon. Another complex area was the liver zone, where sometimes the separation between the colon and the liver is not clear enough to determine, accurately, the separation between both anatomical structures. The use of the synchronized navigation helped the specialist to be confident in this decision.

After experimenting with this new module, some future functionalities were discussed in order to improve their use. Medical experts agree that a useful functionality could be the synchronized correction in both sequences. In this way, the interactive corrections should be only performed in one sequence to fix both colon segmentations simultaneously.

## 4. Discussion

The computerized analysis of MRI images using the proposed algorithm has provided objective information on the volume and distribution of colonic content in normal conditions and in relation to abdominal symptoms [5]. Previous validation studies compared images before and after colonic infusion of known volumes of air, which proved the reliability and precision of the algorithm [13]. Further refinements and automatization of the algorithm have permitted large clinical studies comparing the effect of different dietary interventions on colonic content [4]. Other studies have applied the algorithm to quantify gas and non-gaseous colonic content in different digestive disorders and obtained unique information on the relation between colonic volumes and abdominal symptoms [17].

We wish to acknowledge several limitations of this study. First, we included in the study participants with normal body mass index or overweight, and the performance of the system in patients outside this range remains to be established; particularly, the accuracy may be reduced in underweight participants with a thin abdominal fat layer. Furthermore, the technique has been applied for the evaluation of different physiological conditions in healthy subjects and in patients with functional digestive disorders, but the applicability in case of organic disorders, such as inflammatory bowel disease, intestinal neuropathy, and postsurgical conditions, has not been validated; for instance, colonic morphology may be burred in case of excessive contractile activity.

Our study may have clinical relevance. Indeed, the quasi-automatic current version of the algorithm has hugely accelerated the segmentation process and reduced the specialist’s effort, while maintaining high accuracy and repeatability. Furthermore, the novel synchronized navigation module facilitates segmentation in those areas where it is difficult to distinguish the colon from other adjacent anatomical structures.

Altogether, the improved platform, with an interactive tool for validation, could be suitable for the measurement of colonic volumes in daily clinical practice in the near future.

## Data Availability

Not applicable.

## Abbreviations

The following abbreviations are used in this manuscript:

MRI	Magnetic resonance imaging
T2	T2-weighted HASTE sequence
T1-FS	T1-weighted VIBE fat-sat sequence

## References

[1] Accarino A., Perez F., Azpiroz F., Quiroga S., Malagelada J. (2008). Intestinal Gas and Bloating: Effect of Prokinetic Stimulation. Gastroenterology.

[2] Accarino A., Perez F., Azpiroz F., Quiroga S., Malagelada J. (2009). Abdominal Distention Results From Caudo-ventral Redistribution of Contents. Gastroenterology.

[3] Barba E., Quiroga S., Accarino A., Monclús E., Malagelada C., Burri E., Navazo I., Malagelada J., Azpiroz F. (2013). Mechanisms of abdominal distension in severe intestinal dysmotility: Abdomino-thoracic response to gut retention. Neurogastroenterol. Motil..

[4] Barber C., Mego M., Sabater C., Vallejo F., Bendezu R.A., Masihy M., Guarner F., Espín J.C., Margolles A., Azpiroz F. (2021). 307 Differential Effects of Western and Mediterranean-Type Diets on Gut Microbiota: A Metagenomics and Metabolomics Approach. Nutrients.

[5] Bendezú R., Mego M., Monclus E., Merino X., Accarino A., Malagelada J., Navazo I., Azpiroz F. (2017). Colonic content: Effect of diet, meals, and defecation. Neurogastroenterol. Motil..

[6] Clarke L., Velthuizen R., Camacho M., Heine J., Vaidyanathan M., Hall L., Thatcher R., Silbiger M. (1995). MRI segmentation: Methods and applications. Magn. Reson. Imaging.

[7] Orellana B., Monclús E., Brunet P., Navazo I., Bendezú Á., Azpiroz F. (2020). A scalable approach to T2-MRI colon segmentation. Med. Image Anal..

[8] Brown R.W., Cheng Y.C.N., Haacke E.M., Thompson M.R., Venkatesan R. (2014). Magnetic Resonance Imaging: Physical Principles and Sequence Design.

[9] Pritchard S.E., Marciani L., Garsed K., Hoad C., Thongborisute W., Roberts E., Gowland P.A., Spiller R.C. (2014). Fasting and postprandial volumes of the undisturbed colon: Normal values and changes in diarrhea-predominant irritable bowel syndrome measured using serial MRI. Neurogastroenterol. Motil..

[10] Nilsson M., Sandberg T.H., Poulsen J.L., Gram M., Frøkjær J.B., Østergaard L.R., Krogh K., Brock C., Drewes A.M. (2015). Quantification and variability in colonic volume with a novel magnetic resonance imaging method. Neurogastroenterol. Motil..

[11] Sandberg T.H., Nilsson M., Poulsen J.L., Gram M., Frøkjær J.B., Østergaard L.R., Drewes A.M. (2015). A novel semi-automatic segmentation method for volumetric assessment of the colon based on magnetic resonance imaging. Abdom. Imaging.

[12] Ceballos Inza V., Monclús Lahoya E., Vázquez Alcocer P.P., Bendezú García Á., Mego M., Merino X., Azpiroz Vidaur F., Navazo Álvaro I. (2017). Semi-automatic colonic content analysis for diagnostic. Proceedings of the EuroVis 2017: Eurographics/IEEE VGTC Conference on Visualization 2017.

[13] Bendezú A., Barba E., Burri E., Cisternas D., Accarino A., Quiroga S., Monclús E., Navazo I., Malagelada J.R., Azpiroz F. (2016). Colonic content in health and its relation to functional gut symptoms. Neurogastroenterol. Motil..

[14] Orellana B., Monclús E., Brunet P., Navazo I., Bendezú A., Azpiroz F. Quasi-automatic Colon Segmentation on T2-MRI Images with Low User Effort. Proceedings of the Medical Image Computing and Computer Assisted Intervention—MICCAI 2018 21st International Conference.

[B15-diagnostics-13-00910] 15.Orellana, B.; Monclús, E.; Brunet, P.; Navazo, I.; Bendezú, Á.; Azpiroz, F. Under review 2023.

[16] Males J., Monclús E., Díaz J., Navazo I., Vázquez P.P. (2020). Interactive framework for the visual exploration of colonic data. Comput. Graph..

[17] Malagelada C., Bendezú R.A., Seguí S., Vitrià J., Merino X., Nieto A., Sihuay D., Accarino A., Molero X., Azpiroz F. (2020). Motor dysfunction of the gut in cystic fibrosis. Neurogastroenterol. Motil..

